# Do Non-Responders to Exercise Exist—and If So, What Should We Do About Them?

**DOI:** 10.1007/s40279-018-01041-1

**Published:** 2018-12-17

**Authors:** Craig Pickering, John Kiely

**Affiliations:** 1 Institute of Coaching and Performance, School of Sport and Wellbeing, University of Central Lancashire, Fylde Road, Preston, PR1 2HE UK; 2 Exercise and Nutritional Genomics Research Centre, DNAFit Ltd, London, UK

## Abstract

It is well established that exercise is an important component in the maintenance of good health, and yet recent studies have demonstrated that a sub-section of individuals experience no significant improvements following an exercise training intervention. Such individuals are commonly termed “non-responders”. However, recently a number of researchers have taken a skeptical view as to whether exercise non-response either exists, or is clinically relevant. Here, we explore the research underpinning exercise response, to determine whether non-response to exercise actually exists. We discuss the impact of measurement error and assessment type on the identification of “non-responders”, and whether such non-response is global- or modality-specific. Additionally, we discuss whether, if non-response to an exercise intervention is meaningful and relevant, certain additional interventions—in the form of increasing exercise intensity, volume, or duration—could be made in order to enhance training adaptations. Consequently, based on our interpretations of the available evidence, we suggest that it is unlikely that global non-responders to exercise exist. Furthermore, we suggest this realization effectively counters the perception that some individuals will not positively respond to exercise, and that in turn, this insight serves to encourage health professionals to create more nuanced, efficacious, and individually-focused exercise prescriptions designed to circumvent and overcome apparent non-responsiveness. Adopting a more individually-adaptive approach to exercise prescription could, subsequently, prove a powerful tool in promoting population health.

## Key Points

**Table:** 

“True” exercise non-response is potentially exaggerated by choice of measurement.
Exercise non-response appears to be mitigated by the changing of training variables, including increases in training volume, duration, and intensity.
As a result, it seems unlikely that an individual would exhibit no positive effects from exercise.

## Introduction—A Brief History of Inter-Individual Variation

Typically, researchers are interested in understanding the mean response to an intervention in order to determine its overall efficacy [1]. For example, when determining the effectiveness of resistance training in improving strength, subjects will undertake a pre- and post-training intervention one-repetition-maximum (1RM) test, with the average improvements reported. Similarly, in randomized controlled trials, the mean pre-post change in the intervention group is compared to the mean pre-post change in the control group, and the effectiveness of the intervention determined. However, whilst sports coaches have long understood there is variation in how their athletes respond to a given training stimulus—and researchers often report such variation through the reporting of standard deviations or standard error—only relatively recently has interest in both quantifying and understanding this individual variation through structured research developed [2].

The initial studies exploring the individual response to aerobic training were published in the mid-1980s. The first recruited ten monozygotic twin pairs to a 20-week endurance training program, with pre- and post-intervention measures of maximal aerobic power and ventilatory aerobic threshold. Whilst training enhanced post-training measures *on average* by between 12% (maximal aerobic power) and 20% (ventilatory aerobic threshold), there was considerable variation, with the magnitude of improvements in maximal aerobic power ranging from 0 to 41% [3]. Subsequent studies replicated these initial findings [4, 5], leading to the development of the HERITAGE family study. Here, 720 subjects underwent a 20-week aerobic training program, completing a battery of pre- and post-intervention tests [6]. Again, the results showed significant individual variation; whilst the mean improvement in maximal oxygen uptake (*V*O_2max_) was 384 ml O_2_, some subjects experienced improvements of over 1000 ml O_2_, and others a reduction in *V*O_2max_. Similarly, whilst the mean heart rate (HR) improvement at a workload of 50 W was 11 beats per minute (bpm), some subjects improved by greater than 40 bpm, whilst others became markedly worse [6]. Similar results have been reported following resistance training. In a classic example, Hubal and colleagues [7] recruited 585 subjects to a 12-week progressive resistance training program. Although the mean 1RM improvement was reported as 54%, changes in 1RM ranged from 0 to + 250%. Similarly, changes in maximal voluntary contraction ranged from − 32 to + 149%, and changes in muscle size from − 2 to + 59%. Such extensive variability has also been reported in other resistance training studies [8–10], and those examining other exercise modalities, such as sprint interval training [11, 12].

As such, there are clear individual variations in post-training adaptations, with some subjects exhibiting no meaningful improvements [6, 13], an outcome that conventionally leads to them being labeled as “non-responders” [13]. Alongside this evidence of no clear improvement from exercise, pooled data from six different studies suggest that around 10% of subjects demonstrate an adverse response to training—i.e., exhibit an increased disease risk—defined as a change greater than twice the technical error of measurement in the negative direction, whilst 7% of subjects exhibit an adverse response in at least two variables [14].

Such findings are potentially problematic. If certain individuals cannot improve—or, more troublingly, worsen—their health or fitness following exercise, the implications could be substantial; for some people, exercise may be ineffective, potentially even increasing their disease risk. The purpose of this article is, therefore, to closely examine whether or not exercise non-response exists, and explore the related question of what, if anything, we can do about it.

## The Terminology Problem: “Non-Responder” Versus “Did Not Respond”

Given the increased interest in individual variation, along with the potential for some subjects to exhibit no [6], or negative [14], responses, the term “non-responder” has increasingly been employed to describe those who fail to exhibit positive change in the measured variable following an intervention [15]. The pejorative connotations implicit in such a term, however, may promote a damaging and misleading perception that exercise is perhaps not universally beneficial. This belief is potentially hugely damaging from a public health perspective, given the well-established and wide-ranging positive effects of regular exercise training on reducing obesity risk [16, 17], enhancing cardiometabolic health [18], increasing function in the elderly [19], improving mental health [20], and reducing the risk of various disease states [21–23]. Accordingly, it is crucial to approach such a term, and its related findings, with a critical eye.

One issue worthy of exploration is whether this observed non-response is modality-specific. Whilst the vast majority of studies reporting exercise non-response focus on a single training modality, such as aerobic [3, 6] or strength training [7–9], some examine exercise response across multiple modalities. In one example, 73 subjects completed both endurance and resistance training interventions in a randomized crossover design, with improvements in peak oxygen consumption (*V*O_2peak_) as the measured outcome [24]. As expected, there was individual variation in *V*O_2peak_ improvements from both the aerobic endurance (mean + 8%, range − 5 to + 22) and resistance training (mean + 4%, range − 8 to + 16) interventions, such that some subjects did not improve with a given training modality. Interestingly, however, subjects exhibiting the lowest magnitude of *V*O_2peak_ response following the aerobic training intervention exhibited a positive *V*O_2peak_ response following the resistance training intervention, lending credence to the possibility that changing exercise modality may eliminate, or at least reduce, exercise non-response. One potential limitation of this study is that each training intervention lasted only 2 weeks, a duration shorter than most training studies. Accordingly, it is possible that identified non-responders might have shown increased responses if the intervention period was over more standard timeframes, such as 6–8 weeks. In a second study, having undertaken a combined aerobic endurance and strength training intervention, a small number of subjects exhibited a negative training response in either *V*O_2peak_ or maximum voluntary contraction (MVC), but, crucially, not in both [25]. Finally, Bonafiglia and colleagues [12] reported that, whilst there were non-responders in terms of *V*O_2peak_, lactate threshold, and HR improvements following both endurance and sprint interval training, no subject was a non-responder to both exercise modalities, and very few were non-responders across all three measures for a single exercise intervention. As such, it appears that non-response may be modality-specific; whilst previous authors have suggested that global non-responders to exercise are likely to exist [13], this is not currently supported by experimental data.

A second, less fully appreciated issue relates to the inherent variability of intra-individual adaptive responses. Specifically, it remains currently unclear as to whether or not training responsiveness, to any given intervention, is a permanent or a temporary phenomenon. In essence, if a given intervention were to be repeated within the same population, would the same sub-set of individuals be identified as non-responders? As exercise intervention studies tend not to be repeated on identical subjects, we cannot definitively answer this question. Additionally, as aspects such as baseline fitness, along with dietary factors, training history, and between-session recovery modify the adaptive response to training [26, 27], the individual taking part in an exercise intervention for a second time is likely not in the same adaptive state as when undertaking the initial exercise intervention—further hampering our ability to derive firmer conclusions. As a result, it is not clear whether exercise non-response to a given stimulus is static and unchangeable, remaining uniform when repeated, or dynamic, with “non-responders” showing increased adaptations when repeating a training program. As such, it may be preferable to label individuals exhibiting no measurable improvement in a given variable as those who “did not respond”, representing our uncertainty as to the time-course of such a label, rather than “non-responders”.

## Exercise Non-Response: Methodological Insights

Given the increased interest in exercise non-response and individual variation, a number of researchers have cast a welcome skeptical eye on the underpinning data [28–31]. When determining whether a subject has responded to training, research designs typically require a pre- and post-intervention test, with the difference between the two test scores determining responsiveness [29]. However, inherent within any measurement are both technical error and random within-subject variation [28, 29]; such confounders are said to represent “false” individual variation [28], potentially leading to the mis-identification of individuals as non-responders. To guard against this, a method to determine “true” individual variation has been proposed, whereby the standard deviations of the intervention group are compared to a control/comparator group, as both groups will have similar measurement error and random within-subject variations [28, 30]. Many of the studies supporting the concept of exercise non-response, particularly with regard to aerobic training, lack such a comparator arm [30]. Accordingly, the “true” occurrence of exercise non-response may be overstated, and is currently unclear.

Furthermore, exercise non-response has no set definition; it can refer to the lack of a clinically meaningful change, the lack of a measurable change, a value above the technical error of the test, or to the lowest set percentage of subjects in terms of response [14, 29, 32, 33]. This obviously makes comparisons between trials difficult, as individuals classed as responders in one trial may be classed as non-responders in another, thereby hampering our discernment of the true rate of non-response.

There is also the potential that the type of evaluation utilized may cause differences in test performance that masquerade as individual response. For example, maximal *V*O_2max_ tests are often used to determine improvements in cardiovascular fitness following training. These tests impose significant physical stress and discomfort, ensuring test performance is modulated by subject motivation [34]. Hence, an individual may have undergone significant physiological adaptations from a training program, but performed poorly on the quantifying test due to motivational, non-physiological reasons. Whilst this individual would have responded positively to training, this improvement would not be reflected in test performance. This obviously has important implications for gene association studies exploring exercise non-response; for example, is a particular single nucleotide polymorphism associated with enhanced improvements in aerobic fitness, or does it merely predispose to greater exercise discomfort tolerance [35]? Such a problem could be overcome by more accurate quantification of volitional exhaustion during the pre- and post-intervention exercise testing. For example, the triangulation of relevant objective and subjective measures could provide a more fine-grained estimation of the true extent of fatigue at the point of voluntary cessation, thereby providing a more accurate indication of whether true exhaustion has occurred. Any such improvements in measurement accuracy may well serve to reduce the likelihood of potential mis-labelling of individuals as non-responders to a specific intervention.

Finally, the selection of tested variables appears to affect the identification of exercise non-responders. Typically, non-responders are identified on the basis of one measure, such as 1RM change or improvements in *V*O_2max_. However, when data on more than one variable are collected, exercise non-response seems to disappear. For example, Scharhag-Rosenberger and colleagues [36] had 18 subjects undergo a year-long aerobic training program. Pre- and post-measures were collected for four variables; *V*O_2max_, resting HR, exercise HR, and individual anaerobic threshold. Ten subjects showed no improvement on at least one variable, but, crucially, every subject improved on at least one metric. Similarly, Churchward-Venne et al. [8] retrospectively analyzed subject data collected following 12–24 weeks of resistance training, exploring changes in lean body mass, type I and II fiber size, chair-rise time, leg press, and leg extension 1RM. Again, there were non-responders for each individual measure, but no one subject exhibited non-response across all measures.

## Should We be Concerned About Exercise Non-Response?

At this point, it appears that individual variation in response to exercise is a normal and natural occurrence. This non-response has a statistical component, with the definition of non-response [29], and the variables measured [36], impacting whether an individual is labelled as a non-responder. However, at a population-wide level, by measuring only a few variables and labelling an individual as a non-responder, we run the risk of taking a reductionist approach to exercise. If we accept that exercise is a “polypill”, exerting a plethora of positive benefits [22, 23], then by focusing on a small number of measures of response, we likely miss the bigger picture; exercise works through so many different pathways and mechanisms, that the chances of an individual exhibiting no single biological benefit is highly unlikely. Additionally, exercise clearly exerts benefits above the physiological, reducing stress and improving mental health [20], as well as serving as a social aid [37].

Nevertheless, some physiological measures appear to be more important than others. Timmons [13] referred to this as a “hierarchy of health benefits”, with improvements in aerobic fitness likely to have a greater bearing on health [38] and longevity [39, 40] than other measures. As such, exercise non-response in these higher-tier aspects is clearly important, as maximizing the responsiveness of larger numbers of individuals to exercise could drive important improvements in population health. Additionally, for those at risk of certain diseases, chasing a response in a specific variable may be important. For example, when aiming to reduce type-II diabetes risk in a cohort of at-risk individuals, we seek reductions in fasting glucose and body mass index (BMI) [41]. In this case, non-response to critical variables, and the targeting of effective exercise interventions to overcome non-response, demand greater attention.

## “Did Not Respond”—Potential Interventions

The measurable differences in the magnitude of adaptations following an exercise training program, if clinically relevant, raise the question “what should we do about it?” Findings from a small number of studies provide potentially important information on how best to mitigate, and potentially eliminate, exercise non-response. The simplest approach would be to undertake the training program for longer; Churchward-Venne and colleagues [8] reported that the longer a resistance training intervention lasted, the less prevalent non-response was, and, after 24 weeks, all subjects exhibited a positive response in at least one outcome measure. Sisson et al. [42] demonstrated that the rate of non-response decreased as exercise volume increased, from 45% at a total training exercise expenditure of 4 kcal/kg per week (the lowest volume) to 19% at 12 kcal/kg per week (the highest training volume). Similarly, Ross and colleagues [43] randomly assigned obese subjects to different exercise protocols over a 24-week intervention; low-intensity, low-volume exercise (180–300 kcal per session at 50% *V*O_2peak_); low-intensity, high-volume exercise (360–600 kcal per session at 50% *V*O_2peak_); or high-volume, high-intensity exercise (360–600 kcal per session at 75% *V*O_2peak_). On average, all groups increased their aerobic fitness, although there were a number of subjects deemed to exhibit no response. Interestingly, there were no non-responders in the high-intensity training group, demonstrating that increasing exercise intensity represents a viable method of reducing exercise non-response. Additionally, in the two low-intensity training groups, the group undertaking higher total volumes had fewer non-responders (18%) compared to the group with the lower volume (39%). Furthermore, Astorino and Schubert [11] reported that, following 2 weeks of low-volume sprint interval training, the frequency of non-response was greater than following prolonged, high-volume high-intensity training, again suggesting that exercise intensity is important. Finally, in a paper entitled “Refuting the myth of non-response to exercise training”, Montero and Lundby [44] reported that exercise non-response is dose dependent, finding that it was more likely to occur in subjects exercising 1–2 times per week than in those exercising 4–5 times per week; indeed, there were no non-responders in this latter group. Furthermore, when the subjects identified as non-responders to the initial exercise intervention underwent a second intervention, identical to the first but with two additional weekly training sessions, there were no non-responders. As such, increasing exercise intensity and/or duration appear to be useful strategies for reducing, or perhaps even eliminating, exercise non-response.

A further option for enhancing training outcomes is changing training modality. Because the molecular pathways and gene networks underpinning adaptations to aerobic and resistance exercise are often distinct (although there can be overlap) [13, 45–48], undertaking exercise-types that an individual can more favorably adapt to holds promise. This has been illustrated by Hautala and colleagues [24], whereby individuals termed non-responders following aerobic training enhanced their cardiovascular fitness following resistance training. Additionally, Bonafiglia and colleagues [12] reported that non-response to either typical endurance training or sprint interval training largely abated when subjects undertook the other exercise intervention.

Finally, by understanding the mediators of inter-individual variation, we may be able to enhance the probability of enhanced outcomes, arising from any given training program, for all individuals. At present, research has illustrated that factors such as genotype, baseline fitness, training history, nutritional intake, psycho-emotional states, between-session recovery, age, weight, and ability to meet prescribed training workloads all influence the magnitude of subsequent adaptive responses to exercise [26, 27, 49, 50]. As such, if an individual exhibits a lower response to exercise, correction of some of these factors, such as implementing enhanced sleep hygiene [51] or moderating background psycho-emotional stressors [52], may enhance subsequent training adaptations. Additionally, there remains the possibility that, as the magnitude of exercise response is partially governed by various molecular drivers [13], and as these drivers are partially genetically determined [2, 53], use of genetic information *may* assist in the selection of more individually-optimal training prescription [27, 54]. The potential influence of genotype on training outcomes is an emerging, currently contentious field, with both early promise [55, 56] and null results [57]. As such, the utility of using genotype to guide training interventions requires further research.

## Conclusion—Do Non-Responders to Exercise Exist?

Based on available evidence, we can conclude that:

1. There is an individual variation in response to exercise, with some subjects experiencing larger improvements than others [6, 10, 24].
2. This individual response is a combination of “true” and “false” variation, with “false” variation referring to both technical measurement error and random within-subject biological variation [26], and “true” variation to real, between-subject differences, comprised of differences in both genotype and individual history, amongst other influencing factors [26, 27, 58].
3. There is often a sub-group of individuals who appear to exhibit either no [6, 10] or a negative [14] response to specific exercise training programs.
4. The extent to which this non-response is “true” or “false” within each study currently remains unclear, as is whether this non-response is static (i.e., the individual will always be a non-responder to that particular exercise training program), or merely a temporary reflection of the adaptive capacity of specific individuals at a given time (i.e., the individual did not respond to that exercise training program, but might if the intervention was repeated).
5. Exercise response is often determined by measurement of one, or at most a small number, of all the potential variables that can typically change with exercise. Thus, just because an individual does not improve their *V*O_2max_ or 1RM with training, this does not mean that they have not derived a multitude of other benefits from exercise, many of which, such as increased social interaction seen in community exercise settings [37], are non-physiological in nature.
6. Increasing the number of measured variables can eliminate the prevalence of exercise non-response [36], as can increasing training volume, intensity, or duration [8, 42–44].

As a result, we might, therefore, be better off stating that people “did not respond” to a particular intervention in a given measure, as opposed to labelling them as “non-responders”, because it seems likely that a different training programme (in terms of intensity, volume, duration, or modality) would elicit a positive response. This is similar to the ideas of Booth and Laye [15], who believed the term “non-responder” should be replaced by “low sensitivity”; in this case, these low-sensitivity individuals merely require increased volumes and/or intensity to drive favourable response. Undoubtedly, this is good news, given the wide-ranging benefits of exercise on health and wellbeing; however, further research is required to identify the optimal way to align individuals to the training type most likely to elicit the greatest adaptations, especially given the limited time many people perceive they have available to exercise, along with concerns about the applicability of increased exercise intensities for all exercisers [59]. Furthermore, future research should focus on identifying those expected to exhibit a lower response to exercise, so that they can be given an alternative, more efficacious training intervention. Such research has the potential to have a huge impact on the health of populations, increasing the health and fitness of time-poor individuals in a more effective manner.

## References

[1] Mann T (2011). ‘Mean response’ disregards the importance of individual variation: commentary. S Afr J Sports Med..

[2] Bouchard C (2012). Genomic predictors of trainability. Exp Physiol..

[3] Prud’Homme D, Bouchard C, LeBlanc C, Landry F, Fontaine E (1984). Sensitivity of maximal aerobic power to training is genotype-dependent. Med Sci Sports Exerc..

[4] Despres JP, Bouchard C, Savard R, Tremblay A, Marcotte M, Theriault G (1984). The effect of a 20-week endurance training program on adipose-tissue morphology and lipolysis in men and women. Metabolism..

[5] Simoneau JA, Lortie G, Boulay MR, Marcotte M (1986). Inheritance of human skeletal muscle and anaerobic capacity adaptation to high-intensity intermittent training. Int J Sports Med..

[6] Bouchard C, Rankinen T (2001). Individual differences in response to regular physical activity. Med Sci Sports Exerc..

[7] Hubal MJ, Gordish-Dressman HE, Thompson PD, Price TB, Hoffman EP, Angelopoulos TJ (2005). Variability in muscle size and strength gain after unilateral resistance training. Med Sci Sports Exerc..

[8] Churchward-Venne TA, Tieland M, Verdijk LB, Leenders M, Dirks ML, de Groot LC (2015). There are no nonresponders to resistance-type exercise training in older men and women. J Am Med Dir Assoc..

[9] Erskine RM, Jones DA, Williams AG, Stewart CE, Degens H (2010). Inter-individual variability in the adaptation of human muscle specific tension to progressive resistance training. Eur J Appl Physiol..

[10] Ahtiainen JP, Walker S, Peltonen H, Holviala J, Sillanpää E, Karavirta L (2016). Heterogeneity in resistance training-induced muscle strength and mass responses in men and women of different ages. Age..

[11] Astorino TA, Schubert MM (2014). Individual responses to completion of short-term and chronic interval training: a retrospective study. PLoS One..

[12] Bonafiglia JT, Rotundo MP, Whittall JP, Scribbans TD, Graham RB, Gurd BJ (2016). Inter-individual variability in the adaptive responses to endurance and sprint interval training: a randomized crossover study. PloS One..

[13] Timmons JA (2010). Variability in training-induced skeletal muscle adaptation. J Appl Physiol..

[14] Bouchard C, Blair SN, Church TS, Earnest CP, Hagberg JM, Häkkinen K (2012). Adverse metabolic response to regular exercise: is it a rare or common occurrence?. PLoS One..

[15] Booth FW, Laye MJ (2010). The future: genes, physical activity and health. Acta Physiol..

[16] Ross R, Dagnone D, Jones PJ, Smith H, Paddags A, Hudson R (2000). Reduction in obesity and related comorbid conditions after diet-induced weight loss or exercise-induced weight loss in men: a randomized, controlled trial. Ann Intern Med..

[17] Slentz CA, Houmard JA, Kraus WE (2009). Exercise, abdominal obesity, skeletal muscle, and metabolic risk: evidence for a dose response. Obesity..

[18] Grace A, Chan E, Giallauria F, Graham PL, Smart NA (2017). Clinical outcomes and glycaemic responses to different aerobic exercise training intensities in type II diabetes: a systematic review and meta-analysis. Cardiovasc Diabetol..

[19] Li R, Xia J, Zhang X, Gathirua-Mwangi WG, Guo J, Li Y (2018). Associations of muscle mass and strength with all-cause mortality among US older adults. Med Sci Sports Exerc..

[20] Cooney G, Dwan K, Mead G (2014). Exercise for depression. JAMA..

[21] Booth FW, Roberts CK, Laye MJ (2012). Lack of exercise is a major cause of chronic diseases. Compr Physiol..

[22] Fiuza-Luces C, Garatachea N, Berger NA, Lucia A (2013). Exercise is the real polypill. Physiology..

[23] Pareja-Galeano H, Garatachea N, Lucia A (2015). Exercise as a polypill for chronic diseases. Prog Mol Biol Transl Sci..

[24] Hautala AJ, Kiviniemi AM, Mäkikallio TH, Kinnunen H, Nissilä S, Huikuri HV (2006). Individual differences in the responses to endurance and resistance training. Eur J Appl Physiol..

[25] Karavirta L, Häkkinen K, Kauhanen A, Arija-Blazquez A, Sillanpää E, Rinkinen N (2011). Individual responses to combined endurance and strength training in older adults. Med Sci Sports Exerc..

[26] Mann TN, Lamberts RP, Lambert MI (2014). High responders and low responders: factors associated with individual variation in response to standardized training. Sports Med..

[27] Pickering C, Kiely J. Understanding personalized training responses: can genetic assessment help? Open Sports Sci J. 2017;10(1).

[28] Atkinson G, Batterham AM (2015). True and false interindividual differences in the physiological response to an intervention. Exp Physiol..

[29] Hecksteden A, Kraushaar J, Scharhag-Rosenberger F, Theisen D, Senn S, Meyer T (2015). Individual response to exercise training—a statistical perspective. J Appl Physiol..

[30] Williamson PJ, Atkinson G, Batterham AM (2017). Inter-individual responses of maximal oxygen uptake to exercise training: a critical review. Sports Med..

[31] Atkinson G, Williamson PJ, Batterham AM (2017). Exercise training response heterogeneity: statistical insights. Diabetologia..

[32] Scharhag-Rosenberger F, Meyer T, Walitzek S, Kindermann W (2009). Time course of changes in endurance capacity: a 1-yr training study. Med Sci Sports Exerc..

[33] Vollaard NB, Constantin-Teodosiu D, Fredriksson K, Rooyackers O, Jansson E, Greenhaff PL (2009). Systematic analysis of adaptations in aerobic capacity and submaximal energy metabolism provides a unique insight into determinants of human aerobic performance. J Appl Physiol..

[34] Noonan V, Dean E (2000). Submaximal exercise testing: clinical application and interpretation. Phys Ther..

[35] Pickering C, Kiely J (2017). Exercise genetics: seeking clarity from noise. BMJ Open Sport Exerc Med..

[36] Scharhag-Rosenberger F, Walitzek S, Kindermann W, Meyer T (2012). Differences in adaptations to 1 year of aerobic endurance training: individual patterns of nonresponse. Scand J Med Sci Sports..

[37] Hanson S, Jones A (2015). Is there evidence that walking groups have health benefits? A systematic review and meta-analysis. Br J Sports Med..

[38] Blair SN, Barlow CE, Paffenbarger RS, Gibbons LW (1996). Cardiovascular disease and all-cause mortality in men and women. JAMA..

[39] Blair SN, Kohl HW, Paffenbarger RS, Clark DG, Cooper KH, Gibbons LW (1989). Physical fitness and all-cause mortality. JAMA..

[40] Kodama S, Saito K, Tanaka S, Maki M, Yachi Y, Asumi M (2009). Cardiorespiratory fitness as a quantitative predictor of all-cause mortality and cardiovascular events in healthy men and women: a meta-analysis. JAMA..

[41] Hu G, Lindström J, Valle TT, Eriksson JG, Jousilahti P, Silventoinen K (2004). Physical activity, body mass index, and risk of type 2 diabetes in patients with normal or impaired glucose regulation. Arch Intern Med..

[42] Sisson SB, Katzmarzyk PT, Earnest CP, Bouchard C, Blair SN, Church TS (2009). Volume of exercise and fitness non-response in sedentary, post-menopausal women. Med Sci Sports Exerc..

[43] Ross R, de Lannoy L, Stotz PJ (2015). Separate effects of intensity and amount of exercise on interindividual cardiorespiratory fitness response. Mayo Clin Proc..

[44] Montero D, Lundby C (2017). Refuting the myth of non-response to exercise training: ‘non-responders’ do respond to higher dose of training. J Physiol..

[45] Baar K (2009). The signaling underlying FITness. Appl Physiol Nutr Metab..

[46] Joseph AM, Pilegaard H, Litvintsev A, Leick L, Hood DA (2006). Control of gene expression and mitochondrial biogenesis in the muscular adaptation to endurance exercise. Essays Biochem..

[47] Cantó C, Jiang LQ, Deshmukh AS, Mataki C, Coste A, Lagouge M (2010). Interdependence of AMPK and SIRT1 for metabolic adaptation to fasting and exercise in skeletal muscle. Cell Metabol..

[48] Egan B, Zierath JR (2013). Exercise metabolism and the molecular regulation of skeletal muscle adaptation. Cell Metab..

[49] Bouchard C, Sarzynski MA, Rice TK, Kraus WE, Church TS, Sung YJ (2010). Genomic predictors of the maximal O_2_ uptake response to standardized exercise training programs. J Appl Physiol..

[50] Sarzynski MA, Ghosh S, Bouchard C (2017). Genomic and transcriptomic predictors of response levels to endurance exercise training. J Physiol..

[51] Simpson NS, Gibbs EL, Matheson GO (2017). Optimizing sleep to maximize performance: implications and recommendations for elite athletes. Scand J Med Sci Sports..

[52] Stults-Kolehmainen MA, Bartholomew JB (2012). Psychological stress impairs short-term muscular recovery from resistance exercise. Med Sci Sports Exerc..

[53] Bouchard C, Rankinen T, Timmons JA (2011). Genomics and genetics in the biology of adaptation to exercise. Compr Physiol..

[54] Pickering C, Kiely J (2017). Can the ability to adapt to exercise be considered a talent—and if so, can we test for it?. Sports Med Open..

[55] Delmonico MJ, Kostek MC, Doldo NA, Hand BD, Walsh S, Conway JM (2007). Alpha-actinin-3 (ACTN3) R577X polymorphism influences knee extensor peak power response to strength training in older men and women. J Gerontol A Biol Sci Med Sci..

[56] Jones N, Kiely J, Suraci B, Collins DJ, De Lorenzo D, Pickering C (2016). A genetic-based algorithm for personalized resistance training. Biol Sport..

[57] Charbonneau DE, Hanson ED, Ludlow AT, Delmonico MJ, Hurley BF, Roth SM (2008). ACE genotype and the muscle hypertrophic and strength responses to strength training. Med Sci Sports Exerc..

[58] Sparks LM (2017). Exercise training response heterogeneity: physiological and molecular insights. Diabetologia..

[59] Biddle SJ, Batterham AM (2015). High-intensity interval exercise training for public health: a big HIT or shall we HIT it on the head?. Int J Behav Nutr Phys Act..

